# An In Vitro Assessment of Marginal Accuracies in Copings Fabricated With Two Dissimilar Alloys: An Original Research Study

**DOI:** 10.7759/cureus.28217

**Published:** 2022-08-20

**Authors:** Prince Kumar, Nikhat Fatima, Ganesh Ramesh, Harsh Priyank, Ankita Shrivastav, Susovan Giri, Hiroj Bagde

**Affiliations:** 1 Department of Prosthodontics, Rama Dental College, Hospital, and Research Centre, Kanpur, IND; 2 Department of Periodontology, Rama Dental College, Hospital, and Research Centre, Kanpur, IND; 3 Department of Prosthodontics, Sree Balaji Dental College and Hospital, Bharath Institute of Higher Education and Research (BIHER), Chennai, IND; 4 Department of Conservative, Endodontics, and Aesthetic Dentistry, Dental College, Rajendra Institute of Medical Sciences, Ranchi, IND; 5 Department of Prosthodontics and Crown and Bridge, New Horizon Dental College and Research Institute, Bilaspur, IND

**Keywords:** coping, porosity, marginal accuracy, investment, supranium, mealloy

## Abstract

Background: Marginal accuracy is one of the serious factors that play a key role in the overall success of prostheses. It is openly associated with marginal activities of microorganisms, which may develop micro-leakage and other problems. Therefore, this in vitro study was conducted to assess marginal accuracies in copings fabricated with two different alloys used in fixed partial dentures.

Materials and Methods: Two popularly used metal alloys, Mealloy (nickel-chromium alloy) (Dentsply India Pvt. Ltd., New Delhi, India) and Supranium (nickel-chromium alloy) (Bombay Precision Alloy Inc., Mumbai, India), were studied. Group 1 has 20 copings of Mealloy; group 2 also has 20 copings of Supranium. Blue inlay wax was used for wax pattern fabrication. All copings were cast and made by similar casting techniques. After adequate seating of copings on metal dies, the marginal difference was assessed under a stereomicroscope at typical intensification. All measurements were noticed and converted to the nearest micron. For each coping sample, four measurements were recorded; however, the means of all four surfaces were taken into account for further analysis.

Results: All interrelated data was processed by statistical analysis using the Statistical Package for the Social Sciences (SPSS) software version 22.0 (IBM Corp., Armonk, NY, USA). The overall mean marginal gap of the samples of group 1 was higher than group 2. For group 1 coping samples, a maximum mean marginal gap of 43.379 was noticed at the buccal surface of the copings. P-value computation revealed non-significant values (0.60). For group 2 coping samples, a maximum mean marginal gap of 41.218 was found at the buccal surface of the copings. The measured value was 41.218. One-way ANOVA analysis showed that the degree of freedom was 132.13 for cumulative comparison, while it was 2.930 and 6.837 for calculations between groups and within groups, respectively. Two-sample t-test assessments revealed a p-value of 0.001 (significant) for group 1 and a p-value of 0.810 (non-significant) for group 2.

Conclusion: The marginal space at the margin of the metal coping and the die was minimum for Supranium and maximum for Mealloy. Also, highly significant values were also identified for the metal samples of Supranium. Additionally, the selection of the perfect metal alloy should be entirely dependent on operator skills and clinical decision-making.

## Introduction

The overall success of any fixed partial denture largely depends upon several factors. The longevity of prostheses is a common clinical expectation of patients and clinicians. Many researchers have studied the factors associated with prosthetic failures [1]. Metal ceramic crowns are most popular worldwide due to their acceptability and esthetic and economical nature. However, with the advancements in material science and technologies, many new materials and methods are being practiced in this modern time. Computer-aided milling and direct metal laser sintering systems are very common examples of it [2,3]. Nickel-chromium and cobalt-chromium metallic alloys are very frequently used for fabricating different intraoral prostheses. Both have their own indications and disadvantages. They are sensibly utilized for fabricating traditional crowns and bridges and metal frameworks of cast partial dentures. Repeated casting or recasting alloys have also been studied comprehensively to save the overall expenditure. Nevertheless, all metal alloys pose several clinical issues [4,5]. All such issues cannot be totally omitted; however, we must try to minimize these. These dilemmas are primarily related to marginal adaptation and superadded infections. Thermal contraction and solidification temperature are very imperative factors during the casting procedure. They are directly associated with the dimensional preciseness of prostheses. Any dimensional shrinkage or expansion is not acceptable in any form since they pose serious issues in the future seating of crown over abutment teeth. Additionally, any deviation from recommended manufacturing parameters can also eventually lead to compromised results. These under-qualities could also be related to porosity. So, the operator should be highly attentive while selecting the perfect alloy for a particular clinical condition. During the literature search, we noticed that there are very few studies that compared two novel metal alloys for marginal fit or accuracies [6,7]. Therefore, this in vitro study was conducted to assess the marginal accuracies in copings fabricated with two different alloys used in fixed partial dentures.

## Materials and methods

Marginal accuracy is one of the critical factors that play a key role in the overall success of fixed partial dentures. It is directly related to the marginal activities of microorganisms, which can lead to micro-leakage and other issues. So, in view of these facts, we decided to perform this study using two commonly used metal alloys. The ultimate intention was to explore the response of these tested metals in marginal areas. Metals are used for all metal as well as porcelain fused to metal crowns. However, in this study, metal copings were fabricated in view of future porcelain fused to metal crowns. All samples were categorized into two study groups of 20 each. The used variants of nickel-chromium alloy were Mealloy (Dentsply India Pvt. Ltd., New Delhi, India) and Supranium (Bombay Precision Alloy Inc., Mumbai, India). All samples were categorized into two study groups of 20 each.

Group 1 consisted of 20 samples of Mealloy, while group 2 has 20 samples of Supranium. For ensuring single or standard specifications in all 40 samples, we used metal dies for making wax patterns. This metal die was fabricated with brass metal having ideal tooth preparation similar to the prepared maxillary first molar tooth. All tapers on all four sides were kept ideal and identical. All major and significant line angles were also maintained at ideal levels. Blue inlay wax was utilized with the help of an electrical carver and an electrical wax pot. All prepared wax patterns were then invested in a standard manner by phosphate-bonded investment material. Manufacturers’ guidelines were followed throughout the study for minimizing delinquencies. Burnout of the wax pattern was attempted using a programmed burnout furnace. All samples were cast and fabricated using a similar casting technique. Since we have used brass metal dies for making standardized wax patterns, these standardized wax patterns were casted single-handedly using a single casting operatory and equipment. The resultant castings were finished by a single operator to obtain a standardized coping. Castings with any kind of casting defect were discarded, and the procedure was repeated for the same. All finished metal copings were then separated as per their groups for further analysis. All copings were finished well and then gently triad for seating over ideal metal dies one by one. After satisfactory seating of copings, the marginal discrepancy was evaluated by stereomicroscope at standard intensification. All data and details were entered into the prefabricated spreadsheet in a logical manner. All measurements were made and converted to the nearest micron. For each coping sample, four measurements were made; however, the means of all four surfaces were taken into consideration for further analysis. All related data was processed using Statistical Package for the Social Sciences (SPSS) software version 22.0 (IBM Corp., Armonk, NY, USA).

## Results

Here, it can be clearly outlined that the overall mean marginal gap of the samples of group 1 was higher than group 2. This finding also stands true when the gap was measured on all surfaces. The mean marginal gap of group 2 samples was slightly lower than that of group 1. Table 1 demonstrates predictable marginal space at the margin of the coping and the die for group 1 coping samples (Mealloy: n=20).

**Table 1:** Predictable marginal space at the margin of the coping and the die (group 1) SD: standard deviation; CI: confidence interval

Group	Sides	Mean	SD	SD error	95% CI	df	P-value
Group 1 (Mealloy) (n=20)	Buccal	43.379	0.387	0.372	1.53	1.0	0.60
	Lingual	41.330	0.837	0.031	1.93	1.0	
	Mesial	42.132	0.456	0.243	1.91	2.0	
	Distal	43.028	0.834	0.603	1.52	1.0	

The maximum mean marginal gap was noticed at the buccal surface of the copings. The measured value was 43.379. The minimum mean marginal gap was noticed at the lingual surface of the copings. The measured value was 41.330. The overall mean average marginal gap was 42.20 in microns. P-value calculation revealed non-significant values (0.60). Table 2 shows the expected marginal space at the margin of the coping and the die for group 2 coping samples (Supranium: n=20).

**Table 2:** Predictable marginal space at the margin of the coping and the die (group 2) SD: standard deviation; CI: confidence interval

Group	Sides	Mean	SD	SD error	95% CI	df	P-value
Group 2 (Supranium) (n=20)	Buccal	41.218	0.834	0.603	1.52	1.0	0.02
	Lingual	40.023	0.309	0.054	1.44	1.0	
	Mesial	38.348	0.211	0.254	1.95	1.0	
	Distal	37.202	0.523	0.324	1.56	2.0	

The maximum mean marginal gap was found at the buccal surface of the copings. The measured value was 41.218. The minimum mean marginal gap was identified at the distal surface of the copings. The measured value was 37.202. Less value on the proximal surface was seen in group 2 copings since Supranium exhibits superior properties of marginal adaptation, particularly at proximal surfaces. The overall mean average marginal gap was 39.162 in microns. P-value calculation discovered significant values (0.02). A significant value (p-value) was seen in group 2 because there were obvious marginal gaps noted on all four surfaces along with high variability of standard deviation on these studied surfaces. Table 3 shows the significant comparison among the studied groups by one-way ANOVA.

**Table 3:** Comparison among the two study groups using one-way ANOVA

Variable	Degree of freedom	Sum of squares (∑)	Mean sum of squares (m∑)	F	Level of significance (p)
Between groups	3	2.930	1.738	2.4	0.005*
Within groups	21	6.837	0.038	0	

The degree of freedom was 132.13 for cumulative comparison, while it was 2.930 and 6.837 for calculations between groups and within groups, respectively. The level of significance for overall comparison was highly significant (0.005). Table 4 illustrates the two-sample t-test assessment of the mean score and standard deviation in both the study groups.

**Table 4:** Two-sample t-test assessment of mean score and standard deviation in both study groups SD: standard deviation

Two-sample t-test	Group 1		Group 2	
	Mean score	SD	Mean score	SD
Buccal	43.379	0.387	41.218	0.834
Lingual	41.330	0.837	40.023	0.309
Mesial	42.132	0.456	38.348	0.211
Distal	43.028	0.834	37.202	0.523
P-value	0.001		0.810	

The measurement of the p-value revealed 0.001 (significant) for group 1 and 0.810 (non-significant) for group 2.

## Discussion

Literature has shown different casting techniques, their clinical indications, and their disadvantages. Marginal fit or gap is an evergreen area of interest for prosthodontists. Lombardas et al. in the year 2000 studied the dimensional preciseness of metal copings fabricated with two different investment regimes. They employed ring and ringless investment systems. Their study results were very crucial from an expansion point of view. They confirmed that the use of a ring during investment affects the overall outcome of the casting procedure, particularly accuracy. They also supported the concept of the ringless procedure for making crowns and bridges. These inferences and recommendations are highly comparable with several other studies [8]. Laurent et al. studied the effects of the metal margin gap on the clinical outcomes of the crowns. They introduced a novel method called the silicon replica method. Their study critically utilized silicon material for the overall assessment of the marginal gap between teeth margin and metal margin. They state also stated that outcomes of various casting techniques do not depend upon the form of silicon material [9]. In a recent study conducted by Son et al., investigators studied the marginal fit and other parameters of traditional metals and zircon. They stressed creating a uniformly acceptable guideline for managing margins and internal fit of the prosthesis [10]. Persson et al. utilized a novel method for checking the marginal gap in fixed partial dentures. They used a laser scanner and a touch probe scanner for estimating the amount of gap present at the finish line region of the abutment teeth. They unanimously concluded and stressed the reliability of optical digitizers. It was very much similar to the traditional mechanical digitization apparatus [11]. Traini et al. also used an innovative technique for the assessment of marginal issues. They experimented with direct laser metal sintering for this purpose. Their results were very striking and carry clinical significance. They confirmed that laser metal sintering is a promising innovation, especially in oral implantology, since it is highly compatible with the elasticity of the alveolus [12]. Tan et al. evaluated the marginal gap in computer-aided design-computer-aided manufacturing (CAD-CAM), titanium, and predictable cast metal crowns. Their results were very significant and must be clinically correlated as and when needed. They found no difference among the marginal openings of the tested techniques [13]. Kim et al. assessed the marginal and interior space of metal crowns made by selective laser sintering equipment. They finally confirmed that the selective laser sintering technique is producing large marginal spaces when compared to the standard technique. However, in clinical settings, both methodologies expressed similar performances [14]. Kalavathi et al. also compared ringed and ringless techniques for their effects on the marginal fit of metal. Their study supported the use of the ringless technique for minimizing the marginal gap and improving the performance of the crowns. Therefore, they recommended the ringless technique for improving efficiency and comfort [15]. Lövgren et al. checked the correlation of cobalt-chromium with internal fit, surface smoothness, and marginal accuracies. Their results were highly comparable with our results since they sincerely studied three casting methods with special emphasis on their surface smoothness and retention. They compared laser sintering and the lost wax technique and concluded that the laser sintering method produces prosthesis with enhanced internal fit with minimum marginal gaps [16]. So, the obvious finding of the study was that the marginal space at the margin of the metal coping and the die was minimum for Supranium and maximum for Mealloy.

As limitation, we used only two alloys. We have just used the traditional metal die method for fabrication, and the sample size was limited. Future studies need to be conducted using advanced and newer metal alloys with larger sample sizes and advanced computerized standardization techniques.

## Conclusions

The marginal gap is an unavoidable clinical phenomenon that usually tends to deteriorate the performance and durability of the prosthesis. However, this clinical issue can be successfully minimized using a few clinical guidelines, biological considerations, and specifications. Within the limitations of the study design, the marginal space at the margin of the metal coping and the die was minimum for Supranium and maximum for Mealloy. Highly significant values were also drawn from the metal samples of Supranium. Although the inference of this study is very striking, we do not intend to promote any particular alloy or system. Furthermore, a precise selection of ideal metal alloy must be dependent on operator skills and clinical decision-making.
